# New records of Protura (Entognatha, Arthropoda) from Romania, with an identification key to the Romanian species

**DOI:** 10.3897/zookeys.552.6613

**Published:** 2016-01-13

**Authors:** Julia Shrubovych, Cristina Fiera

**Affiliations:** 1 Institute of Systematics and Evolution of Animals, Polish Academy of Sciences, Sławkowska 17, Pl–31016, Kraków, Poland; 2 State Museum of Natural History, Ukrainian National Academy of Sciences, Teatral'na St. 18, UA 79008, L'viv, Ukraine; 3 Institute of Biology Bucharest of Romanian Academy, 296 Splaiul Independenţei, P.O. Box 56-53, 060031, Bucharest, Romania

**Keywords:** Protura, Romania, distribution, ecology, remarks, identification key

## Abstract

The Romanian Protura were studied based on 175 specimens collected from Romania, along with bibliographic data. The main publication on the Romanian proturans was written by M.A. Ionescu (1951), who described 13 species mainly from soil and forest litter from 15 collecting points. The current paper represents the first study at a national level. Faunal data on Protura were obtained from 22 sites, mostly from forests of the Romanian Carpathians and also from a peri-urban area of Bucharest, which had not been studied before. As a result, the Romanian Protura fauna now consists of 27 known taxa in 6 genera and 4 families. Of the 27 taxa, 15 species are new records for Romanian fauna. An identification key to the Romanian Protura species is provided.

## Introduction

Proturans are found world-wide except in the Arctic and Antarctic regions (Szeptycki 2007) and primarily live in soil, leaf litter, mosses, and decaying wood. These minute soil-inhabiting hexapods also can be collected from animal burrows, meadows, and agriculture soils or urban areas.

The first report on Romanian Protura was that of Ionescu (1930) who described five species (*Acerentomon robustum* Ionescu, 1930, *Acerentomon mesorhinus* Ionescu, 1930, *Acerella muscorum* (Ionescu, 1930), *Acerentulus aureus* Ionescu, 1930 (= *Acerentulus confinis* Berlese, 1908)) and *Paraentomon carpaticum* (now *Ionescuellum carpaticum* Ionescu, 1930 (Tuxen 1960)), and reported two other species: *Eosentomon semiarmatum* Denis, 1927 and *Eosentomon transitorium* Berlese, 1909 from forest humus at Sinaia-Cumpătul, 850 m elevation. To date, 13 species of Protura have been reported from Romania (Ionescu 1951). All of these records originated from soil and forest litter samples from 15 collecting sites. One species, *Acerentomon robustum* Ionescu, 1930, was established as “species inquirenda" (Szeptycki 2007) because the species was insufficiently described and type material was lost (Tuxen 1961). We have not taken into account this species in the present paper. Falcă (1972) identified four species of Protura from the Retezat Mountains from three types of forests along an elevational gradient of 850‒1800 m.

The fauna of Romanian proturans is poorly known in contrast to some other European countries: Luxembourg, 30 species in 10 genera (Szeptycki et al. 2003); Poland, 69 species of Protura in 11 genera (Szeptycki 2007); Ukraine, 58 species in12 genera (Shrubovych 2010); Austria, 58 species in 10 genera (Christian 2011); Italy, 40 species in 8 genera (Galli et al. 2011); Serbia, 38 species in 10 genera (Blesić and Mitrovski-Bogdanović 2012).

The aim of this study is to improve the study of this little known taxon in Romania by providing new records and distributional data on proturan species.

## Materials and methods

**Figure 1.**
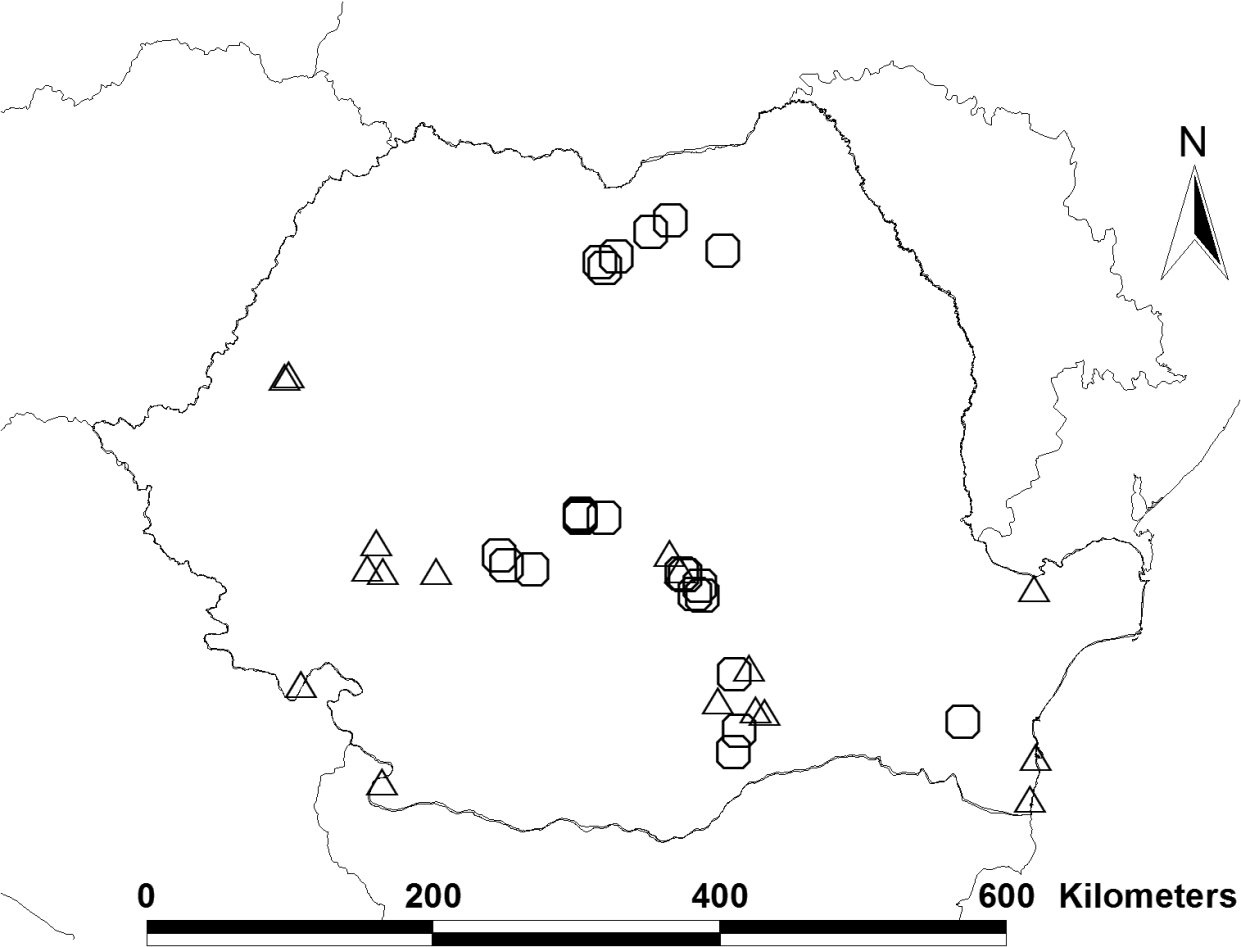
Locations of Protura collection sites in Romania. Octagons indicate collections studied by authors; triangles represent sites recorded by Ionescu (1930, 1932, 1937, 1951) and Falcă (1972).

Protura were extracted from samples of leaf litter, soil and mosses in Berlese funnels. The material has been deposited in the Institute of Systematics and Evolution of Animals, Polish Academy of Sciences, Kraków (ISEA). Specimens were mounted on slides in Marc Andre medium and were observed and identified with a phase-contrast microscope. In total 175 specimens from 22 sampling sites were examined. In our analysis we also considered the data taken from 17 Romanian collecting sites mentioned in the literature. Species were identified based on a key to European Protura (Nosek 1973) and other papers (Shrubovych et al. 2012, 2014, Szeptycki 1980, 1985, 1986, 1991). The taxonomic system of Protura presented by Szeptycki (2007) was followed in this paper. Species distributions were taken from Szeptycki (2005, 2007), Shrubovych (2010) for Ukraine, Galli et al. (2011) for Italy and Blesić and Mitrovski-Bogdanović (2012) for Serbia. All collecting sites are shown in Fig. 1.

### Abbreviations

The following abbreviations are used throughout the paper: pi – preimago, mj - maturus junior, LII - larva II, LI - larva I.

Chaetal nomenclature follows Nosek (1973) and Szeptycki (1986): *A*-setae – anterior setae, *P*-setae – posterior setae, aa and ap-setae on head – anterior and posterior additional setae, respectively.

### List of sampling sites in Romania

Ilfov County: Periş, 44°40'18"N; 26°1'44"E, elevation 100 m, mixed forest, soil, 06. XI.2012.

Ilfov County: Jilava, 44°19'00.038"N; 26°03'54.579"E, elevation 50 m, mixed forest, soil, 02. XI.2013.

Prahova County: Valea Largă, 45°18'20.638''N; 25°34'19.943''E, elevation 770 m, beech forest, in dead trunk, 13.XI.2013.

Prahova County: Şotriile, 45°13'39''N; 25°43'44''E, elevation 600 m, located on the mountainside above the Doftana River, mountain beech forest with *Luzula luzuloides* (Lam.) Dandy and Wilmott, soil and litter, 12.XI.2013.

Prahova County: Voila, 45°09'58''N; 25°45'10''E, elevation 500 m, *Quercus petraea* (Mattuschka) Liebl. and *Fagus sylvatica* L. mixed forests, soil and litter, 12.XI.2013.

Prahova County: Cheile Brezei: 45°10'31.906"N; 25°41'16.153"E, elevation 455 m, shrubs and soil on rocks, 11.IX.2014.

Prahova County: Cheile Posadei, 45°17'39.947"N; 25°36'00.001"E, elevation 720 m, mosses and litter on rocks, 12.XI.2013.

Suceava County: Adam Peak, 47°30'58.17"N; 25°29'09.09"E, elevation 950 m, spruce forest, mosses on soil, 26.VIII.2014.

Suceava County: Iacobeni, 47°26'45.913"N; 25°18'41.182"E, elevation 915 m, *Larix decidua* Mill., soil, 26.VIII.2014.

Făgăraş Mountains: Valea Arpaşului, 45°40'52"N; 24°40'12"E, elevation 685 m, beech forest with *Abies alba* Mill. and *Acer* sp., soil, 10.IX.2014.

Făgăraş Mountains: Valea Arpaşului, 45°39'21.937"N; 24°40'13.930"E, elevation 850 m, forest with *Abies alba* and *Fagus sylvatica* L., soil, 10.IX.2014.

Făgăraş Mountains: Valea Arpaşului, 45°40'01.337"N; 24°40'15.289"E, elevation 820 m, harvested forest with *Abies alba*, soil, 10.IX.2014.

Făgăraş Mountains: Valea Brescioarei, 45°39'16.773"N; 24°53'09.207"E, elevation 930 m, spruce forest, 31.VII.2014.

Bârgău Mountains: Lunca Ilvei, 47°19'37.267"N; 25°58'20.189"E, elevation 785 m, forest with *Abies alba* and *Fagus sylvatica*, soil, 28.VIII.2014.

Bârgău Mountains: Piatra Fântânele, 47°17'27.107"N; 24°59'45.294"E, elevation 915 m, spruce forest, soil, 28.VIII.2014.

Bârgău Mountains: Valea Străjii, 47°13'05.330"N; 24°53'36.495"E, elevation 800 m, beech forest mixed with spruce, soil, 29.VIII.2014.

Bârgău Mountains: Tureac, 47°15'26.614"N; 24°50'54.282"E, elevation 795 m, meadow, soil, 29.VIII.2014.

Vălcea County: Voineasa, 45°25'00"N; 23°57'20"E, elevation 705 m, beech forest, soil, 01.XI.2012.

Cozia National Park: Călineşti Valley near Brezoi, 45°19'48"N; 24°14'30"E, elevation 610 m, litter of beech forest mixed with *Pinus* sp., 21.X.2012.

Olt Valley: Malaia, 45°21'11.18"N, 24°01' 11.74' E, elevation 495 m, beech forest, litter near rocks, 01.XI.2012.

Giurgiu County: Călugăreni, 44°10'48.839"N, 26°00'42.400"E, elevation 70 m, mixed forest, soil, 03.XI.2013.

Constanţa County: Cernavoda, 44°20'11.92'N, 28°01'05.211''E, elevation 55 m, shrubs, soil, 10.XI.2012.

## Results

Twenty-two species of Protura were identified in this study based on our sampling material and 15 new records for Romanian proturan fauna were established. In total, 27 species belonging to 6 genera and 4 families (Hesperentomidae, Protentomidae, Acerentomidae and Eosentomidae) are now recorded from Romania.

Some data about ecology, distribution in Europe and in Romania are given for each species and, where appropriate, remarks are provided. An identification key to the Romanian Protura species follows the species accounts.

### Order Acerentomata

#### Family Hesperentomidae

##### Subfamily Hesperentominae

**1. *Ionescuellum carpaticum* (Ionescu, 1930)**

**Ecology.** This species prefers dry to moderately humid rock mountain biotopes (Nosek 1973).

**Distribution in Romania.** This species was hitherto known only from Sinaia, Prahova County; lives under rocks in the forest (Ionescu 1930).

**Distribution in Europe.** Austria (Szeptycki 2005); Bosnia and Herzegovina, Croatia, Greece, Poland (Szeptycki 2007); Serbia (Blesić and Mitrovski-Bogdanović 2012).

#### Family Protentomidae

##### Subfamily Protentominae

**2. *Proturentomon minimum* (Berlese, 1908)**

**Material examined.** Site 1, 2 females.

**Ecology.** Usually occurs in grasslands (Nosek 1973); peri-urban forest (in present study).

**Distribution in Romania.** This species is known only from Periş forest (Ilfov County) (this study).

**Distribution in Europe.** Austria, Bosnia and Herzegovina, Czech Republic, Italy, Germany, Great Britain, Greece, Luxembourg, Poland, Portugal, Switzerland, Slovakia, European Russia (Szeptycki 2007); Serbia (Blesić and Mitrovski-Bogdanović 2012).

**Remarks.** New record for the Romanian fauna.

**3. *Proturentomon* sp.**

**Material examined.** Site 3, one female.

**Remarks.** Probably a new species, more material is needed for description. This species has four anterior setae on tergites II‒VI (*A1* and *A2*), whereas 10 *Proturentomon* species have only two anterior setae (*A1*) and three species lack them entirely.

#### Family Acerentomidae

##### Subfamily Berberentulinae

**4. *Acerentulus alni* Szeptycki, 1991**

**Material examined.** Site 8, 2 females; site 3, 3 females; site 4, 3 females, 2 mj; site 10, one male, 1 pi; site 11, one female, 1 mj; site 13, 2 females, one male, 1 pi, 4mj; site 15, 2 females, one male 1LII.

**Ecology.** Lives in various types of forests and meadows (Szeptycki 1991, Shrubovych 2010).

**Distribution in Romania.** Adam Peak, Prahova County: Valea Largă, Şotriile, Făgăraş Mountains: Valea Arpaşului and Valea Brescioarei, Bârgău Mountains (this study).

**Distribution in Europe.** Poland, Slovakia (Szeptycki 2007) and Ukraine (Shrubovych 2010).

**Remarks.** New record for the Romanian fauna.

**5. *Acerentulus confinis* (Berlese, 1908)**

**Ecology.** Eurytopic species; previously recorded from soil, litter and mosses of both humid and xerothermic forests, in garden soil, in mosses on limestone rocks, in detritus, and along the Black Sea coast (Nosek 1973).

**Distribution in Romania.** Sinaia (Prahova County) and in Pantelimon forest, near Bucharest (Ilfov County), in litter; Agigea (Constanţa County), in litter of *Crataegus* bushes, on dunes from zoological station at Agigea (Ionescu 1951).

**Distribution in Europe.** Austria, Belgium, Bosnia and Herzegovina, Great Britain, Bulgaria, Corsica, Czech Republic, France, Germany, Greece, Hungary, Ireland, Italy, Portugal, Slovakia, Switzerland (Szeptycki 2007); Canary Islands, Madeira; Poland and Ukraine (Shrubovych 2006, Szeptycki 2007); Serbia (Blesić and Mitrovski-Bogdanović 2012); doubtful in: Balearic Islands, Slovenia, Spain (Szeptycki 2005).

**6. Acerentulus cf. confinis**

**Material examined.** Site 18, one female, one male.

**Remarks.** Probably a new species or an intrapopulation variation. Our species differs from *Acerentulus confinis* (Berlese, 1908) in the absence of setae *P3a* on tergite VII and in a longer foretarsus (135 µm versus 100 µm in *Acerentulus confinis*).

**7. *Acerentulus exiguus* Condé, 1944**

**Material examined.** Site 5, one female; site 10, one male; site 12, 3 females, one male; site 13, one female.

**Ecology.** Eurytopic species; abundant in soil, litter, mosses, decaying wood and plant-debris of forests, meadows, xerothermic grasslands and shrubs on rocks (Szeptycki 1991, Shrubovych 2010).

**Distribution in Romania.** Voila forest (Prahova County), Făgăraş Mountains: Valea Arpaşului and Valea Brescioarei (this study).

**Distribution in Europe.** Greek mainland and Ukraine (Szeptycki 2005); Austria, Bosnia and Herzegovina, Corsica, Czech Republic, France, Germany, Poland, Sardinia, Slovakia (Szeptycki 2007); Serbia (Blesić and Mitrovski-Bogdanović 2012).

**Remarks.** New record for the Romanian fauna.

**8. *Acerentulus halae* Szeptycki, 1997**

**Material examined.** Site 6, one female, one male.

**Ecology.** Xerophilous species; the species was found in plant debris in meadow-steppes (Shrubovych 2010).

**Distribution in Romania.** This species was reported only from Adam Peak (this study).

**Distribution in Europe.** Known only from Ukraine (Szeptycki 2007).

**Remarks.** New record for the Romanian fauna.

**9. *Acerentulus traegardhi* Ionescu, 1937**

**Material examined.** Site 6, one male.

**Ecology.** Xerophilous species; abundant in soil and litter of forests, meadow-steppes and green patches inside cities squares (Szeptycki 1991, Shrubovych 2010).

**Distribution in Romania.** Comarova, near Black Sea, south of Agigea (Constanţa County), litter in forest (Ionescu 1937); Retezat Mountains, litter and humus of three sampling sites: 1) *Festuco drymejae*-*Fagetum* community, elevation 850 m; 2) *Hieracio transilvanico*-*Piceetum* community, elevation 1250 m; 3) *Calamagrostio villosae*-*Pinetum mugo* community, elevation 1800 m (Falcă 1972).

**Distribution in Europe.** Austria, Belgium, Bosnia and Herzegovina, Bulgaria, Denmark, France, Germany, Greece, Hungary, Ireland, Italy, Poland, Slovakia, Spain, Sweden and Ukraine; some records from Europe have been misidentified as *Acerentulus insignis* and should be confirmed, especially from western Europe (Szeptycki 2007); Serbia (Blesić and Mitrovski-Bogdanović 2012).

**10. *Acerentulus xerophilus* Szeptycki, 1979**

**Material examined.** Site 9, 2 females, 2 males.

**Ecology.** Xerophilous species; reported from soil and litter of forests, meadow-steppes, dry grasslands and city squares (Szeptycki 1991, Shrubovych 2010).

**Distribution in Romania.** This species is known only in Iacobeni (Suceava County) (this study).

**Distribution in Europe.** Poland and Ukraine (Szeptycki 2007, Shrubovych 2010), Serbia (Blesić and Mitrovski-Bogdanović 2012).

**Remarks.** new record for the Romanian fauna.

**11. Acerentulus sp. cunhai -group**

**Material examined.** Site 20, one female.

**Remarks.** The Romanian specimen belongs to the *cunhai*-group according to Nosek's criteria (1973). It differs from other members of the group in absence of seta *P1a* on tergites II-III and possession of this seta on tergites IV-V. This specimen probably represents a new species, but more material is necessary.

**Distribution in Romania.** Malaia (Olt Valley) (this study).

**Remarks.** New record for the Romanian fauna.

##### Subfamily Acerentominae

**12. *Acerentomon affine* Bagnall, 1912**

**Material examined.** Site 7, one female, one male.

**Ecology.** This species prefers forest biotopes (Nosek 1973).

**Distribution in Romania.** Calafat (Dolj County) and Ciocăneasa, in forest litter (Ionescu 1951).

**Distribution in Europe.** Austria, Bosnia and Herzegovina, Great Britain, France, Germany, Ireland, Luxembourg, Spain and Sweden (Szeptycki 2007), Italy (Galli et al. 2011).

**13. *Acerentomon carpaticum* Nosek, 1961**

**Material examined.** Site 3, one female, 1 mj, 2 LII; site 16, 1 mj, site 17, 5 females, 2 males.

**Ecology.** This species prefers forest biotopes (Nosek 1973, Shrubovych 2006).

**Distribution in Romania.** Valea Largă (Prahova County); Bârgău Mountains (Valea Străjii and Tureac) (this study).

**Remarks.** New record for the Romanian fauna.

**Distribution in Europe.** Ukraine (Shrubovych 2006); Bosnia and Herzegovina, Poland and Slovakia (Szeptycki 2007).

**14. *Acerentomon mesorhinus* Ionescu, 1930**

**Ecology.** Reported from forest and meadow biotopes (Shrubovych 2006).

**Distribution in Romania.** Cumpatul - Sinaia (Prahova County, beech forest (Ionescu 1930); Retezat Mountains, litter and humus of three sampling sites: 1). *Festuco drymejae*-*Fagetum* community, elevation 850 m; 2) *Hieracio transilvanico*-*Piceetum* community, elevation 1250 m; 3) *Calamagrostio villosae*-*Pinetum mugo* community, elevation 1800 m (Falcă 1972).

**Distribution in Europe.** Germany, Slovakia, Ukraine; Serbia (Blesić and Mitrovski-Bogdanović 2012).

**15. *Acerentomon microrhinus* Berlese, 1909**

**Ecology.** This species prefers forest biotopes (Nosek 1973, Shrubovych 2006).

**Distribution in Romania.** Parang Mountains, forest humus, 1000 m elevation, and in litter of oak forest in Pantelimon, near Bucharest (Ilfov County) (Ionescu 1951).

**Distribution in Europe.** Austria, Bosnia and Herzegovina, Corsica, France, Italy, Slovakia, Slovenia and Ukraine (Szeptycki 2007), Serbia (Blesić and Mitrovski-Bogdanović 2012).

**16. *Acerentomon quercinum* Ionescu, 1932**

**Material examined.** Site 20, one female, 1 mj.

**Ecology.** Prefers forest biotopes (Nosek 1973, Szeptycki 1980, Shrubovych 2006).

**Distribution in Romania.** Forest humus, Cernica (Ilfov County) and litter of oak forest, Ciocăneasa (Ionescu 1951); Retezat Mountains, litter and humus of three sampling sites: 1) *Festuco drymejae*-*Fagetum* community, elevation 850 m; 2) *Hieracio transilvanico*-*Piceetum* community, elevation 1250 m; 3) *Calamagrostio villosae*-*Pinetum mugo* community, elevation 1800 m (Falcă 1972).

**Distribution in Europe.** Austria, Bosnia and Herzegovina, Hungary, Slovakia, Poland and Ukraine (Szeptycki 2007); data from France should be confirmed (Szeptycki 2007); Serbia (Blesić and Mitrovski-Bogdanović 2012).

**17. Acerentomon cf. quercinum**

**Material examined.** Site 19, 3 males, 4 females, 1 mj.

**Remarks.** These specimens probably represent an undescribed species. Acerentomon cf. quercinum differs from *Acerentomon quercinum* Ionescu 1932 in having foretarsal sensillum *a* shorter, and maxillary and labial sensilla with a different shape; in our opinion these characters are not sufficient for description of a new species. Molecular analysis could clarify their status.

**18. *Acerentomon rostratum* Ionescu, 1951**

**Material examined.** Site 18, 2 females, 4 males, 1 LII, 1 LI.

**Ecology.** Lives in oak forest (Ionescu 1951).

**Distribution in Romania.** Bumbeşti-Piţic, Copaci forest, oak forest (Gorj County) (Ionescu 1951).

**Distribution in Europe.** Known only from Romania (Szeptycki 2007).

##### Subfamily Acerentominae

**19. *Acerella muscorum* (Ionescu, 1930)**

**Ecology.** This species prefers forest ecosystems (Nosek 1973, Shrubovych 2006, 2010).

**Distribution in Romania.** Sinaia-Cumpătul (Prahova County), in mosses (Ionescu 1930); beech forest and in litter of oak forests from Tăgădău and Galaleu (Arad County) (Ionescu 1951).

**Distribution in Europe.** Germany (Szeptycki 2005), Austria, Bosnia and Herzegovina, Bulgaria, Czech Republic, France, Greece, Hungary, Italy, Poland, Sardinia, Slovakia, Spain, Switzerland and Ukraine (Szeptycki 2007), Serbia (Blesić and Mitrovski-Bogdanović 2012).

### Order Eosentomata

#### Family Eosentomidae

**20. *Eosentomon armatum* Stach, 1926**

**Material examined.** Site 9, 2 mj; sit 21, 2 females.

**Ecology.** In soil and litter of forests (beech-hornbeam and oak) (Szeptycki 1985), found also in urban parks (Shrubovych 2006).

**Distribution in Romania.** Ponicova (Cazane) (Mehedinți County), in forest humus; in mosses on soil in Sinaia (Prahova County); Snagov (Ilfov County), Săbăreni, Tăgădău and Galaleu (Arad County) and under bark of Jepi, at 2000 m elevation near Caraiman Peak, Bucegi Mountains (Ionescu 1951).

**Distribution in Europe.** Austria, Belgium, Czech Republic, Denmark, France, Great Britain, Iceland, Germany, Luxembourg, Poland, Portugal, Slovakia, Spain, Switzerland and Ukraine. All data before 1986 needs verification as some records are mixed with similar species (Szeptycki 2007), Italy (Galli et al. 2011).

**21. *Eosentomon carpaticum* Szeptycki, 1985**

**Material examined.** Site 4, 2 females; site 5, 2 females, one male, 1 LI; site 7, one male, 1 mj; site 14, 1 mj; site 19, one female, 2 males; site 20, one female.

**Ecology.** Found in soil and moss in beech forest and under dense overgrown shrubs (Szeptycki 1985, Shrubovych 2006).

**Distribution in Romania.** Şotriile, Cheile Posadei and Voila (Prahova County); Bârgău Mountains: Lunca Ilvei; Cozia National Park: Călineşti Valley; Olt Valley: Malaia (this study).

**Distribution in Europe.** Endemic Carpathian species, Ukraine and Poland (Szeptycki 2007).

**Remarks.** New record for the Romanian fauna.

**22. *Eosentomon enigmaticum* Szeptycki, 1986**

**Material examined.** Site 3, one female, 2 males, 1 LII.

**Ecology.** This species prefers forest ecosystems (Szeptycki 1986).

**Distribution in Romania.** Known only from Valea Largă (Prahova County) (this study).

**Distribution in Europe.** Poland and Ukraine (Shrubovych 2006, Szeptycki 2007).

**Remarks.** New record for the Romanian fauna.

**23. *Eosentomon pinetorum* Szeptycki, 1984**

**Material examined.** Site 1, 4 females, one male; site 4, one male; site 10, 3 females; site 12, 1 mj.

**Ecology.** Xerophilous species; lives in beech, oak, hornbeam, pine forests and mixed forests with spruce, thermophilous fir forest and steppe localities (Szeptycki 1998, Shrubovych 2010).

**Distribution in Romania.** Periş (Ilfov County); Şotriile (Prahova County); Făgăraş Mountains: Valea Arpaşului (this study).

**Distribution in Europe.** Austria, Czech Republic, Germany, Poland and Ukraine (Shrubovych 2006, Szeptycki 2007).

**Remarks.** New record for the Romanian fauna.

**24. *Eosentomon semiarmatum* Denis, 1927**

**Material examined.** Site 22, 3 females.

**Ecology.** Eurytopic species; reported from different forest and steppe localities (Szeptycki 1986, Shrubovych 2006).

**Distribution in Romania.** Forest litter from Sinaia (Prahova County); Cernica (Ilfov County) and Snagov (Ilfov County) (Ionescu 1951).

**Distribution in Europe.** Balearic Islands, France, Germany, Poland and Ukraine (Szeptycki 2007).

**25. *Eosentomon silvaticum* Szeptycki, 1986**

**Material examined.** Site 3, one male, 1 LII.

**Ecology.** The species prefers woodlands (fir and mixed forests with fir, pine, beech, hornbeam) (Szeptycki 1986).

**Distribution in Romania.** This species is known only from Valea Largă (Prahova County) (this study).

**Distribution in Europe.** Poland and Luxembourg (Szeptycki 2007).

**Remarks.** New record for the Romanian fauna.

**26. *Eosentomon stachi* Rusek, 1966**

**Material examined.** Site 2, 3 females, one male, 2 mj.

**Ecology.** Xerophilous species; lives in different forests, meadows, dry pasture ground and in petrophilous turf on limestone (Szeptycki 1985, Shrubovych 2006).

**Distribution in Romania.** This species is known only from Jilava forest (Ilfov County) (this study).

**Remarks.** New record for the Romanian fauna.

**Distribution in Europe.** Austria, Luxembourg, Poland, Slovakia and Ukraine (Szeptycki 2007).

**27. *Eosentomon transitorium* Berlese, 1908**

**Material examined.** Site 5, one female, 1 mj; site 7, one female; site 11, one male, 1 LII.

**Ecology.** Eurytopic species; it has been recorded from soil and litter of various forests, town parks, alpine bushes *Salix herbacea* L. and *Dryas octopelata* L., in debris of tall herbs on rock shelves, dry meadows, grasslands, in xerothermic turf, in deep soil under stones (Nosek 1973, Szeptycki 1986, Shrubovych 2006).

**Previous records**: Sinaia (Prahova County), in beech litter; Agigea, near zoological station (Constanţa County), in litter, *Acacia* forest (Ionescu 1951); Retezat Mountains, litter and humus of three sampling sites: 1) *Festuco drymejae*-*Fagetum* community, elevation 850 m; 2) *Hieracio transilvanico*-*Piceetum* community, elevation 1250 m; 3) *Calamagrostio villosae*-*Pinetum mugo* community, elevation 1800 m (Falcă 1972).

**Distribution in Europe.** Latvia, Estonia; most of these records should be confirmed (Szeptycki 2005); Austria, Bosnia and Herzegovina, Czech Republic, Denmark, Finland, France, Great Britain, Greece, Hungary, Germany, Italy, Ireland, Norway, Poland, Slovakia, Sweden, Switzerland and Ukraine; doubtful records from: Balearic Islands, Belgium, Bulgaria, Corsica, Croatia, Iceland, Portugal, Sardinia, Spain (Szeptycki 2007), Serbia (Blesić and Mitrovski-Bogdanović 2012).

## Key to Romanian Protura

**Table:** 

1	Spiracles present on meso- and metanota (Eosentomata, *Eosentomon*)	**2**
–	Spiracles absent (Acerentomata)	**9**
2	Head setae *aa* and *ap* present, notal setae *P2a* and *P3a* of equal length (*Eosentomon delicatum*-group)	**3**
–	Head setae *aa* absent, *ap* present, notal seta *P2a* shorter than *P3a* (*Eosentomon transitorium*-group)	**5**
3	Foretarsal sensillum *c*' on the line α*6*-δ*5*	***Eosentomon carpaticum***
–	Foretarsal sensillum *c*' proximal to the line α*6*-δ*5*	**4**
4	Sensillum *c*' broadened, *P1a* on tergite VIII without basal dilation, notal seta *P2a* longer than *P3a*	***Eosentomon stachi***
–	Sensillum *c*' slender, *P1a* on tergite VIII with basal dilation, notal seta *P2a* the same length as *P3a*	***Eosentomon armatum***
5	Seta *P1a* on tergite VII at the same level as *P2*	**6**
–	Seta *P1a* on tergite VII posterior to level of *P2*	**7**
6	Seta *D2* on metatarsus slender	***Eosentomon transitorium***
–	Seta *D2* on metatarsus spine-like	***Eosentomon enigmaticum***
7	Sternites IX–X with 6 setae	***Eosentomon pinetorum***
–	Sternites IX–X with 4 setae	**8**
8	Seta *P1a* on tergites II–IV longer than *P1*, foretarsal sensillum *t3* longer than *c*'	***Eosentomon semiarmatum***
–	Seta *P1a* on tergites II–IV shorter than *P1*, foretarsal sensillum *t3* same length as *c*'	***Eosentomon silvaticum***
9	Abdominal appendages I–II two-segmented	**10**
–	Abdominal appendage I two-segmented, appendage II not segmented (Acerentomidae)	**12**
10	Tergites II–VII with 4 pairs of *A*-setae, calyx of maxillary gland small	***Ionescuellum carpaticum*** (**Hesperentomidae)**
–	Tergites II–VII with at most 2 pairs of *A*-setae, calyx of maxillary gland enlarged (Protentomidae)	**11**
11	Tergites II–VI with one pair of *A*-setae, foretarsal sensillum *b* about 2/3 length of *c*	***Proturentomon minimum***
–	Tergites II–VI with 2 pairs of *A*-setae, foretarsal sensillum *b* nearly as long as *c*	***Proturentomon* sp.**
12	Meso- and metanota with 2 pairs of *A*-setae, abd. appendages II–III each with 3 setae…14	***Acerentulus***
–	Meso- and metanota with 3 or 4 pairs of *A*-setae, abd. appendages II–III each with 2 setae	**13**
13	Meso- and metanota both with 3 pairs of *A*-setae, foretarsal sensillum *t1* filiform, calyx of maxillary gland with racemose appendices	***Acerella muscorum***
–	Mesonotum with 3 pairs of *A*-setae, metanotum with 4 pairs of *A*-setae, sensillum *t1* claviform, calyx of maxillary gland smooth (*Acerentomon*)	**21**
14	Sternite XI with 4 setae	**15**
–	Sternite XI with 6 setae	**16**
15	Foretarsal sensillum *b* very long, nearly reaching the base of claw, tergite VII with seta *P3a*	***Acerentulus traegardhi***
–	Foretarsal sensillum *b* shorter, nearly equal to length of sensillum *c*, tergite VII without seta *P3a*	***Acerentulus halae***
16	Foretarsal sensillum *a* short, passing the base of sensillum *t2* (*Acerentulus cunhai*-group)	***Acerentulus* sp.**
–	Foretarsal sensillum *a* long, nearly reaching or surpassing the base of seta γ*3* (*Acerentulus confinis*-group)	**17**
17	Tergite VII with 3 pairs of *A*-setae	**18**
–	Tergite VII with 4 pairs of *A*-setae	**19**
18	Tergite VII with seta *P3a*, sternite VII with seta *Pc*	***Acerentulus xerophilus***
–	Tergite VII without seta *P3a*, sternite VII without seta *Pc*	***Acerentulus exiguus***
19	Tergite VII with seta *P3a*	**20**
–	Tergite VII without seta *P3a*	**Acerentulus cf. confinis**
20	Maxillary sensilla spindle-shaped, foretarsal sensillum *a*' slender, sternites II-III without pores	***Acerentulus confinis***
–	Maxillary sensilla parallel-sided and slender, foretarsal sensillum *a*' thickened, sternites II-III with pores	***Acerentulus alni***
21	Tergite VII with pair of *x*-setae(*Acerentulus doderoi*-group)	***Acerentomon rostratum***
–	Tergite VII without *x*-setae	**22**
22	Sternite VIII with a pair of posterior setae (*Acerentomon affine*-group) ***Acerentomon affine***
–	Sternite VIII without posterior setae (23)	***Acerentomon microrhinus*- group**
23	Labrum slightly protruded (LR = 9), foretarsal sensilla *a*, *b* and *c* of the same length	***Acerentomon microrhinus***
–	Labrum clearly protruded (LR = 7 or less), sensilla *a*, *b* and *c* of unequal lengths	**24**
24	Foretarsal sensillum *b* broadened	**25**
–	Foretarsal sensillum *b* slender	**26**
25	Foretarsal sensillum *a* long, reaching the base of seta γ*3*, maxillary sensilla spindle-shaped, labial sensillum slender	***Acerentomon quercinum***
–	Foretarsal sensillum *a* short, slightly surpassing the base of seta γ*2*, maxillary sensilla parallel-sided, labial sensillum broadened	**Acerentomon cf. quercinum**
26	Foretarsal sensillum *a* longer than *c*, sternite VI with 5 *A*-setae	***Acerentomon carpaticum***
–	Foretarsal sensillum *a* shorter than c, sternite VI with 7 *A*-setae	***Acerentomon mesorhinus***

## References

[B1] Blesić B Mitrovski-Bogdanović A (2012) Protura in Serbia. Kragujevac Journal of Science 34: 101‒106. http://www.pmf.kg.ac.rs/KJS/images/volumes/vol34/kjs34blesicmitriovski101.pdf

[B2] Christian E (2011) Protura (Insecta). In: Schuster R (Ed.) Checklisten der Fauna Österreichs, No 5: Protura (Insecta), Opiliones (Arachnida), Pseudoscorpiones (Arachnida), Tipulidae (Insecta: Diptera). Biosystematics and Ecology Series, Austrian Academy of Sciences Press 28: 1‒9.

[B3] Falcă M (1972) Species of Protura from scientific reservation: Retezat National Park. Studii şi comunicări, 95‒99. [In Romanian]

[B4] Galli L Capurro M Torti C (2011) Protura of Italy, with a key to species and their distribution. ZooKeys 146: 19‒67. doi: 10.3897/zookeys.146.1885 22207788 10.3897/zookeys.146.1885 PMC3233705

[B5] Ionescu MA (1930) Note sur quelques Protoures de Sinaia (Roumanie). Bulletin de la Section scientifique de l'Académie Roumaine 13(1-2): 1‒9.

[B6] Ionescu MA (1932) Contributions to study of the fauna of beech litter. Statistical, ecological and systematics research of beech forest from Sinaia and Prahova Valley. Bucharest, 100 pp [In Romanian]

[B7] Ionescu MA (1937) La chaetotaxie des stades larvaires chez le genre *Eosentomon* (Ord. Protura). Entomologisk Tidskrift 58: 101‒105.

[B8] Ionescu MA (1951) Insecta Fascicula 1 Protura. In: Fauna Republicii Populare Române, vol. 7 Academia Republicii Populare Române, Bucureşti 7(1): 1‒38.

[B9] Nosek J (1973) The European Protura. Their taxonomy, ecology and distribution with keys for determination. Museum d'Histoire Naturelle, Geneve, 345 pp.

[B10] Shrubovych JJ (2006) Catalogue of Protura species. In: Kaprus' IJ Shrubovych JJ Tarashchuk MV (Eds) Catalogue of the Collembola and Protura of Ukraine. State Natural History Museum NAS of Ukraine, Lviv, 126‒135.

[B11] Shrubovych J (2010) Taxonomical richness and chorological structure of Protura fauna of Ukraine. Scientific Bulletin of the Uzhgorod University, Seria Biologia 29: 75‒81.

[B12] Shrubovych J Schneider C D‘Haese C (2012) Description of a new species of *Acerentulus* Berlese, 1908 (Protura: Acerentomata: Acerentomidae) with its barcode sequence and a key to the *confinis* group. Annales de la Société Entomologique de France 48(1-2): 1‒7. doi: 10.1080/00379271.2012.10697746

[B13] Shrubovych J Schneider C D‘Haese C (2014) Two new species of *Acerentulus* Berlese, 1908 (Protura: Acerentomata: Acerentomidae) with its barcode sequence and a key to the *cunhai* group. Annales de la Société Entomologique de France 50(2): 129‒140. doi: 10.1080/00379271.2014.934036

[B14] Szeptycki A (1980) Polish Protura I. Genus *Acerentomon* Silvestri, 1907. Polskie Pismo Entomologiczne 50: 311‒392.

[B15] Szeptycki A (1985) Polish Protura II. *Eosentomon delicatum* Gisin, 1945 and related species. Polskie Pismo Entomologiczne 55: 531‒574.

[B16] Szeptycki A (1986) Polish Protura IV. *Eosentomon* „*transitorium*" group. Polskie Pismo Entomologiczne 56: 481‒530.

[B17] Szeptycki A (1991) Polish Protura V. Genus *Acerentulus* Berlese, 1908 (Acerentomidae). Acta Zoologica Cracoviensia 34: 1‒64.

[B18] Szeptycki A (2005) Protura. In: Fauna Europaea, version 2.6.2. [last updated on 29 August 2013 by Shrubovych J] http://www.faunaeur.org [accessed 05 August 2015]

[B19] Szeptycki A (2007) Catalogue of the world Protura. Acta Zoologica Cracoviensia 50B: 1–210. http://www.ingentaconnect.com/content/isez/azcb/2007/00000050/00000001/art00001

[B20] Szeptycki A Stomp N Weiner WM (2003) The Protura of Luxembourg. Ferrantia 34: 5‒44.

[B21] Tuxen SL (1960) Eine neue Gattung von Proturen: *Ionescuellum*. Videnskabelige Meddelelser fra dansk naturhistorisk Forening i Kjøbenhavn 123: 21‒32.

[B22] Tuxen SL (1961) Neues über die von Ionescu beschriebenen Proturen (Apterygota). Beiträge zur Entomologie 11: 281‒329.

